# A proposed novel digital condylar position adjustment technique to help restore a normal disc-condyle relationship

**DOI:** 10.1016/j.heliyon.2024.e32037

**Published:** 2024-05-31

**Authors:** Yanji Gong, Fang Liu, Yunfan Zhu, Qinlanhui Zhang, Jinyi Zhu, Yang Liu, Deqiang Yin

**Affiliations:** a National Clinical Research Center for Oral Diseases, West China Hospital of Stomatology, Sichuan University, Chengdu, 610041, China; b College of Medicine, Xi'an International University, Xi'an, 710077, China; c College of Aerospace Engineering, Chongqing University, Chongqing, 400044, China

**Keywords:** Temporomandibular joint (TMJ), Disc-condyle relationship, Occlusal transfer, Condyle position

## Abstract

**Objectives:**

To demonstrate a novel digital technique that enables real-time visualisation of occlusal transfer and adjustment of condyle position, to (1) improve the repeatability of occlusal transfer and the accuracy of condyle position adjustment and (2) be clinically effective in helping to restore the disc-condyle relationship.

**Materials and methods:**

Three participants were included in the study and underwent facebow transfers using two different methods. The digital method used patient-related three-dimensional imaging data matched with digital dental casts for occlusal transfer. The conventional method used anatomical facebows. The condylar position was adjusted based on occlusal transfer results. The results were evaluated and compared in three dimensions. In addition, clinical application data from 36 patients were analysed before and after condylar position adjustment. Statistical significance was set at p < 0.05.

**Results:**

Differences in the spatial positions of the three anatomical structures reproduced by both methods were statistically significant (p = 0.000). After adjusting the rotation of the condylar position, the positional deviation of the condylar point along the X- and Z-axes was significantly lower in the digital group (p < 0.05). After adjustment for translation (X and Z), the positional deviation showed no difference along the X- and Z-axes (p > 0.05) but a significant difference along the Y-axis (p < 0.001).

**Conclusion:**

A novel digital technique for occlusal transfer and condylar position adjustment was presented. This technique simplifies clinical practice, improves the accuracy of results, and can help restore a normal disc-condyle relationship.

## 1 Introduction

The temporomandibular joint (TMJ) should be considered while formulating a dental treatment plan [[1], [2], [3]]. Altering the condylar position by adjusting the positional relationship between the maxillary and mandibular dentition can affect mandibular movements, and an optimal position of the condyles in the articular fossa may improve mandibular function and facial asymmetry and establish a normal disc-condyle relationship [4,5]. Therefore, recording and adjusting the positions of the condyle and mandible are important in treatment. This requires the use of a facebow and an articulator to record the patient's occlusal relationship, which is called occlusal transfer. The spatial relationship of the maxilla and mandible can be recorded and adjusted in vitro [6]. The clinical importance of the use of facebows is controversial because there is no evidence to support that their use leads to better clinical outcomes [7].

Several approaches have been documented for ascertaining an accurate occlusal transfer [8]. The kinematic facebow dynamically traces mandibular movements through pin-pad plotting measurements, ultrasonic induction, or photoelectric induction to virtually calculate and plot the individual hinge axis [[9], [10], [11], [12], [13], [14], [15]]. The anatomical facebow was designed using an abstractive simulation of the kinematic facebow. It uses superficial anatomical landmarks to locate the possible hinge axis, which, based on statistical analyses, may be positioned 11–13 mm anterior to the ear tragus along the Frankfort Horizontal plane [16]’.

However, these methods estimate the location of the functional hinge axis based on randomly recorded mandibular movements or soft tissue landmarks, instead of recording the actual bony structure, which may result in low repeatability and reproducibility of the condyle and hinge axis locations, high variability, and a lack of correspondence with anatomy [[17], [18], [19], [20], [21], [22], [23]]. In clinical practice, accuracy of records made using anatomical facebows can be significantly influenced by the skill level of the operator. As a result, the transferred dentition on the articulator may not be a precise map of the actual spatial position of the mandible [[24], [25], [26], [27], [28], [29]]. In addition, avoiding inaccuracies associated with the use of materials in traditional methods such as plaster expansion and deformation of bite-registration materials is difficult [30].

Since digital technology is widely used in dentistry, process simplification and the precise designing and implementation of condylar position adjustment can be accomplished based on digital techniques [31]. The use of virtual facebows has become increasingly popular for creating virtual patients from data obtained from facial scans, computed tomography (CT) scans, and dental models of actual patients [32,33]. However, facial scanners are not readily available in clinics, and the accuracy of the scans is questionable because they are based on soft-tissue imaging. An in vitro study showed that the selection of different facial scanning methods has a strong impact on the accuracy of three-dimensional (3D) facial localisation [34]. Therefore, a technique that accurately maps the patient's actual structure and simulates the condylar position for better occlusal transfer and condylar position adjustment, which can simplify time-consuming clinical procedures, is required.

The current study introduces a novel digital condylar position adjustment technique that enables real-time visualisation of occlusal transfer and adjustment of condylar position. The aims of the new technique are (1) to improve the repeatability of occlusal transfer and the accuracy of condylar position adjustment, and (2) to be clinically effective in restoring the disc-condyle relationship. This study compared the consistency of the new technique workflow with the conventional workflow in terms of reproduction of the position of three structures (maxillary dentition, bilateral condyles, and mandible) after performing five occlusal transfers and evaluated the deviation of the condyle after adjusting the condylar position based on the results of each occlusal transfer to assess the accuracy and validity of the adjustments. In addition, this study evaluated the clinical application data (condyle and articular disc displacement, articular disc morphology, and disc-condyle relationship) before and after using the digital technique to adjust the condylar position in 36 patients to assess the effectiveness of the technique.

## 2 Material and methods

### Ethics approval

This study was approved by the Institutional Review Board of the West China Hospital of Stomatology (approval number: WCHSIRB-D-2022-208-R1).

All participants provided informed consent to participate in the study and for the publication of their anonymised case details and images.

### 2.1 Selection of participants

**Fig. 1:**
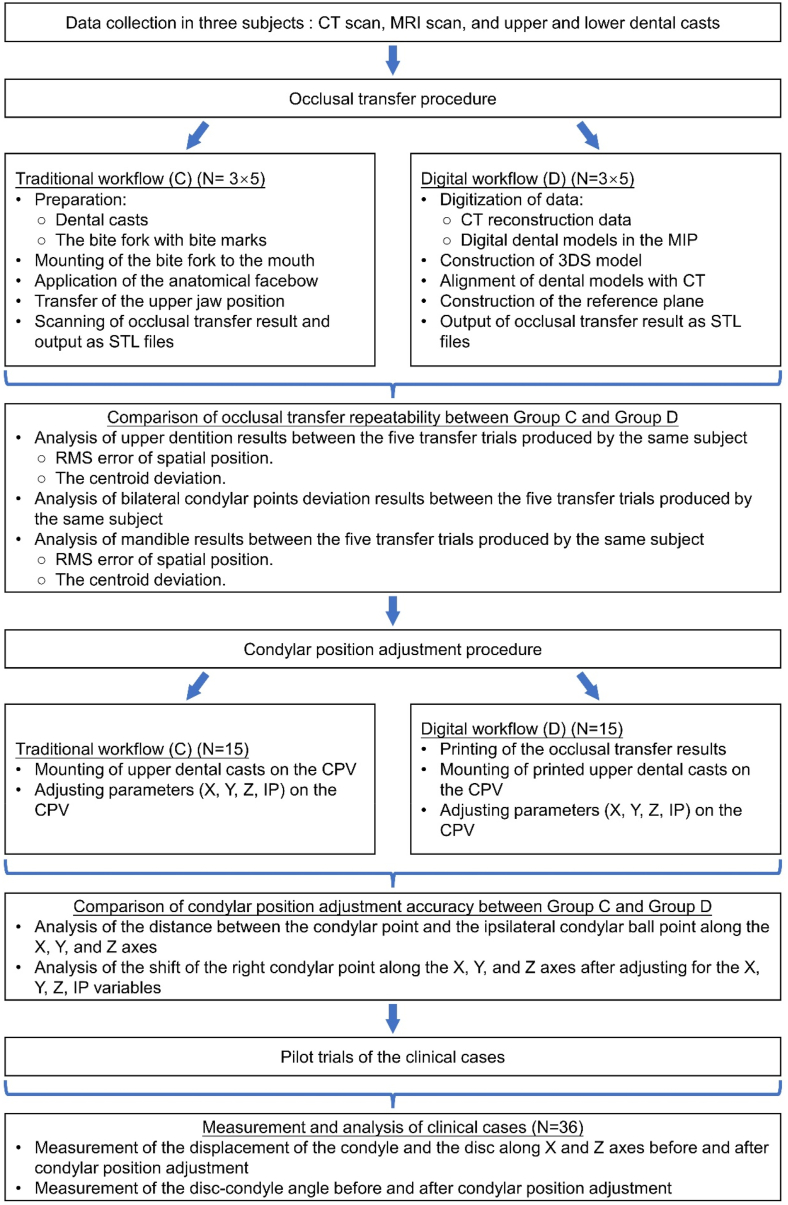
Schematic diagram of the experimental workflow. MRI, magnetic resonance imaging; STL, standard tessellation language; MIP, maximum intercuspation position; 3DS, three-dimensional stomatognathic; RMS, root mean square; CPV, condyle position variator

Three subjects were recruited according to the following inclusion criteria: (1) permanent and complete dentition with a normal overjet and overbite, (2) no jaw deformities, and (3) no symptoms, signs, or history of TMJ disorders. Each participant underwent a digital and conventional workflow, as shown in Fig. 1. The participants underwent CT scanning (including the entire mandible and maxilla) with teeth in the maximum intercuspation position (MIP) and magnetic resonance imaging (MRI) with teeth in the MIP and mandible in maximum opening position, as previously described. Maxillary and mandibular dental models were made [5]. The above data were collected for subsequent experimental procedures.

In addition, CT and MRI data were collected from 36 clinical patients according to the inclusion and exclusion criteria in whom condylar position was adjusted with the digital technique. Informed consent was obtained from all patients. The inclusion criteria were as follows: (1) complete and clear initial craniomaxillofacial CT and bilateral TMJ MRI data, (2) age ≥11 years, and (3) substantially intact dentition. The exclusion criteria were as follows: (1) unclear or partially or completely missing craniomaxillofacial CT or bilateral TMJ MRI data, (2) history of orthodontic or orthoprosthetic surgical treatment, (3) severe dental or periodontal disease (≥3 teeth affected), and (4) history of cleft lip or palate or other systemic diseases affecting craniofacial growth and development. The condyle and articular disc displacement, articular disc morphology, and disc-condyle relationship were assessed and compared before and after adjustment [35].

### 2.2 The occlusal transfer procedure

**Fig. 2:**
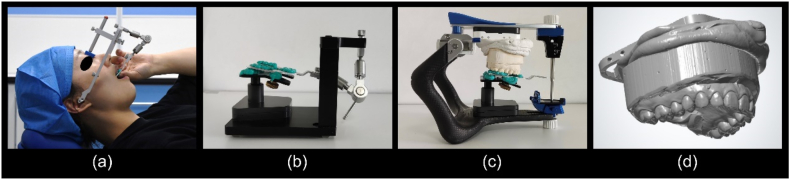
Steps in occlusal transfer using an anatomical facebow. (a) Mount the anatomical facebow with the bite fork on the patient. (b) Fix the bite fork registration on a plaster bed on the transfer table. (c) Transfer the maxillary jaw position to the articulator. (d) Scan the maxillary dentition with the split mounting plate.

In the control group (C), five occlusal transfers were performed for each participant using an anatomical facebow (Artex Facebow; Amann Girrbach, Koblach, Austria). For each trial, the respective maxillary dental cast was affixed to the split mounting plate on the articulator (Artex CR; Amann Girrbach, Koblach, Austria) using gypsum according to the conventional method [36] (Fig. 2a–c). The maxillary dental cast with the split mounting plate was scanned using a laboratory scanner (DS-EX Pro (C); SHINING 3D, China); data were exported in the standard tessellation language (STL) format for subsequent analysis (Fig. 2d). Two experienced TMJ specialists performed occlusal transfers in the same participant using the conventional method.

In the digital group (D), the complete workflow of the digital technique was used and five trials were performed for each participant for comparison with the control group. The digital technique was used as follows.

**Fig. 3:**
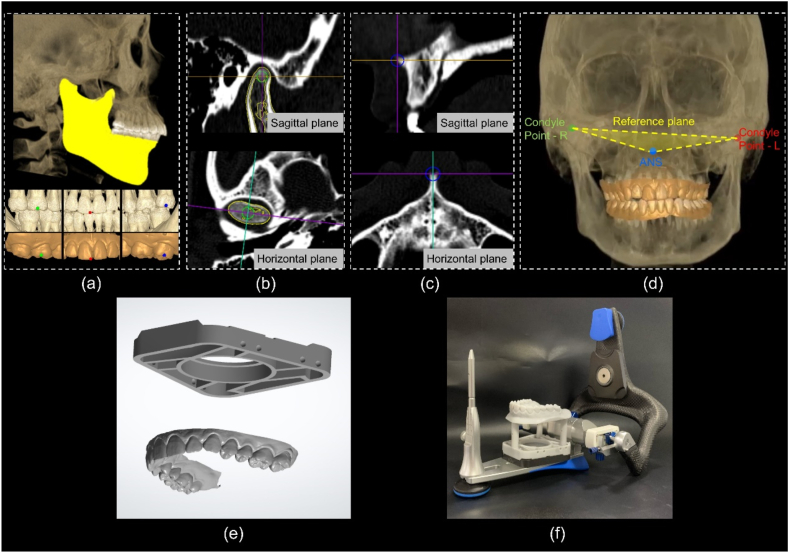
Steps in occlusal transfer using the digital technique. (a) Extract the mandible from the three-dimensional stomatognathic (3DS) model (highlighted in yellow), match the maxillary dentition by selecting three anchor points. (b) Define the right condylar point (same for the left) as the centroid centre of the condyle. (c) Define the anterior nasal spine (ANS). (d) Build the reference plane (highlighted in yellow) using the ANS and right and left condylar points, in relation to which the dental models are located. (e) (f) The digital articulator split mounting plate connected with the maxillary dentition was designed, printed, and mounted on the articulator.

The CT reconstruction data of each participant were extracted into digital imaging and communications in medicine format to construct a 3D stomatognathic (3DS) model. The mandibular and maxillary dentitions were extracted and reconstructed separately (Fig. 3a). The maxillary and mandibular dental casts were scanned in the MIP using a laboratory scanner, and the data were exported in STL format. The same three anchors (preferably morphologically characterised for easy recognition) were selected on the maxillary dentition of the 3DS model and the digital maxillary dentition. By aligning the anchor points, the 3DS model was matched with the digital dental model and the position was fine-tuned as needed. The mandibular dental model was imported directly in the MIP with the maxillary dental model and matched with the mandible of the 3DS model, as both scans were taken at the same position (MIP) (Fig. 3a). Three reference points were defined on the 3DS model to set up the reference plane and coordinates: the bilateral condylar points (the fitting centres of the working surface on the bilateral condyles) and the anterior nasal spine (ANS) (Fig. 3b–d). A digital articulator split mounting plate connected to the maxillary dentition was added to the corresponding spatial position of the reference plane, printed, and mounted on the articulator (Fig. 3e–f) [37].

**Fig. 4:**
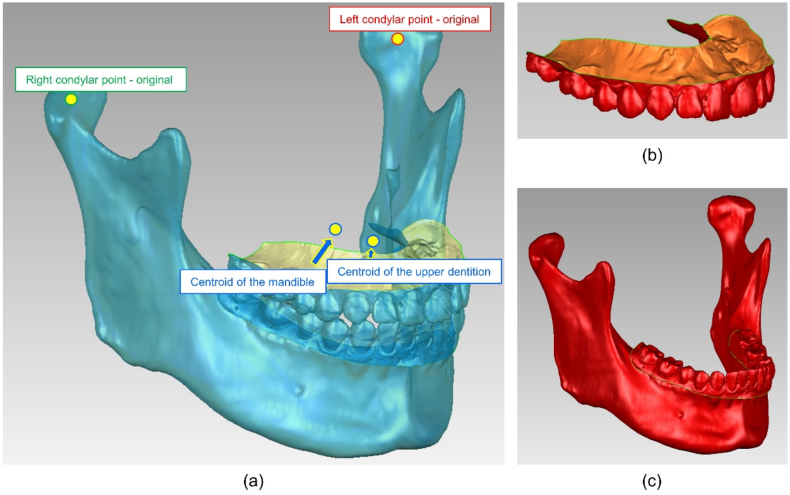
Schematic representation of the reference model created in the Geomagic software. (a) Definition of the left and right condylar points, centroid of the maxillary dentition, and centroid of the mandible using the ‘creating centroid’ tool. (b) The area of the maxillary dentition selected for analysis. (c) The area of mandible selected for analysis.

For each participant, a 3DS model was constructed, and the mandible was extracted based on CT data using the Mimics Research software (Version 20.0; Materialize, Leuven, Belgium). By setting the threshold at ‘Bone (CT)’, the mandible was isolated to build a 3DS model in an STL format. The surface of the model was smoothed using the Geomagic Wrap program (Version 2021; Raindrop, North Carolina, America). The scanned dental casts were aligned and merged with the mandibular model using the ‘N-point alignment’ and ‘best-fit alignment’ tools. A reference model was used for subsequent analyses. The geometric centre for the right or left condyle working surface was defined as the condylar point defined using the ‘creating centroid’ tool (Fig. 4a). After the occlusal transfers, five maxillary dental models were superimposed by selecting their split mounting plates using the ‘N-point alignment’ and ‘best-fit alignment’ tools in the Geomagic Wrap software program and their spatial positions were unified. Subsequently, the 3DS reference model was matched to each maxillary cast by aligning the maxillary dentition such that the mandibular dentition and the mandible were located based on the scanned MIP relationship. Finally, the spatial positions of the mandible and condyles were determined after each transfer.

The repeatability of the occlusal transfers was first assessed. Using the Geomagic Wrap program, the following three sites were selected for positional deviation analysis: the dentition was isolated along the gingival margin, mandibular body, and bilateral condyles (Fig. 4a–c). The positional shift between the five transfer trials was evaluated for any two models in the two groups, and 30 sets of comparisons were obtained. The root mean square (RMS) error was calculated by obtaining colour difference maps using the ‘3D compare’ tool; the smaller the RMS value obtained, the better the 3D matching of the superimposed data and therefore, the smaller the positional shift [38,39]. The linear distance between the condylar points on each side was measured using the ‘measuring the distance from a feature’ tool.

### 2.3 The condylar position adjustment procedure

Adjustment of the condylar position was based on the result of each occlusal transfer and performed using a fully adjustable articulator (CPV; GAMMA DENTAL, Austria) [40]. Because the CPV can adjust the condylar position in three axes, X, Y, and Z, it was considered. For the digital group, a 3D model was printed based on each exported STL file, which could be magnetically mounted on the CPV. A mandibular dental cast was mounted on the CPV in the MIP with the maxillary dentition. For the control group, dental casts were mounted on the CPV. Adjustment was performed according to parameter combinations of 1, 2, and 3 mm per adjustment in the X, Y, and Z axes and 1, 2, 3, 4, and 5° per adjustment for the incisal pin (IP) variable.

**Fig. 5:**
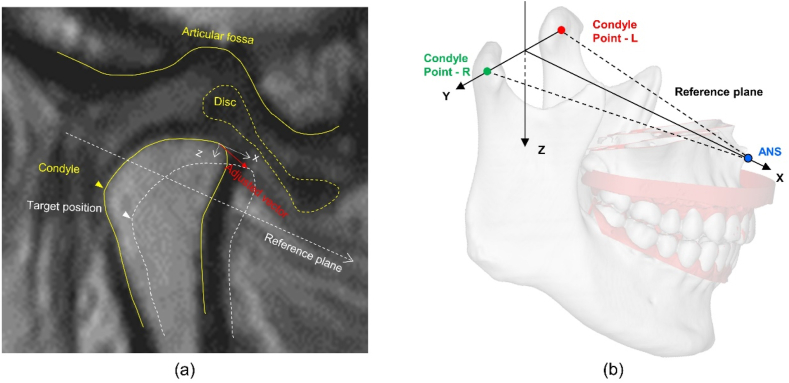
Adjustment strategy for condylar position. (a) The lateral condyle can be moved to the target position by adjusting the X, Y, and Z and incisal pin (IP) values. (b) The Y axis was defined by connecting the right and left condylar points, with its positive direction toward the right. The X axis was defined as perpendicular to the Y axis along the reference plane, with its positive direction toward the front. The Z axis was defined as perpendicular to the reference plane, with its positive direction downward. Each condylar point is allowed to translate along the X, Y, and Z axes. The mandible can rotate around the Y axis, and the IP variable can be adjusted to simulate opening and closing in the vertical dimension.

The accuracy of condylar position adjustment was subsequently assessed. For each adjustment trial, the spatial relationship between the maxillary and mandibular dentition was scanned and consolidated using the 3DS reference model. A reference coordinate system was established based on the 3DS model, the distance between the condylar point and the ipsilateral condylar ball point was measured along the X, Y, and Z axes, and the shift of the right condylar point along the X, Y, and Z axes after adjusting for the X, Y, Z, and IP variables was measured using the ‘measuring the distance from a feature’ tool (Fig. 5a and b).

### 2.4 Pilot trials of the clinical cases

The CT and MRI data from 36 patients who underwent condylar position adjustments using the digital technique in clinical practice were analysed using the Mimics software. After the digital occlusal transfer, further adjustments to the condylar position were made using the digital technique. The use of this digital technique facilitates visualisation and precise design of the jaw position, aiding in accurate adjustments throughout the process.

**Fig. 6:**
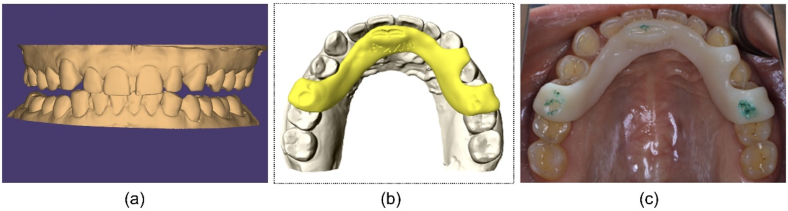
Condylar position adjustment can be optionally conducted with real time visualisation. (a) The mandibular digital dental model registered as the three-dimensional stomatognathic (3DS) model can also achieve the target position as the condylar position is adjusted. (b) (c) The bite guiding splint was designed and 3D printed.

After adjustment, the spatial positions of the maxillary and mandibular dentitions in relation to the reference plane was exported in separate STL files, with the parameters exported separately to the record pad file (Fig. 6a). A three-point bite-guiding splint was designed and fabricated using 3D printing (Fig. 6b). During try in, on recording the bite, the mandible was guided into the pre-designed position, as was the disc-condyle relationship, and the patient's appearance was be changed due to the mandible position shift (Fig. 6c and Supplementary Video).

**Fig. 7:**
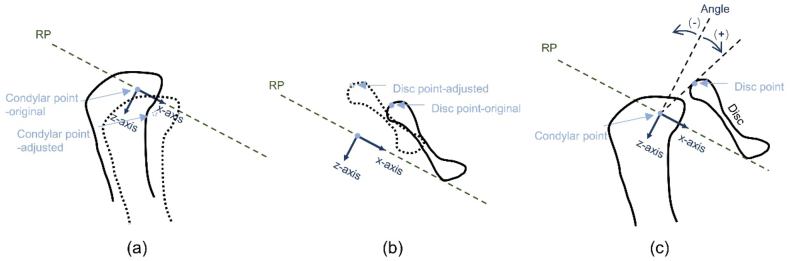
The condylar and disc movements and the disc-condyle angle measurements. (a) The displacement of the condylar point-adjusted from the condylar point-original was measured after adjusting the condylar position. (b) The displacement of the disc point-adjusted from the disc point-original was measured after adjusting the condylar position. (c) The disc-condyle angle was measured before and after adjusting the condylar position. RP: Reference plane.

The displacement of the condyle and disc along the X and Z axes and the disc-condyle angle before and after condylar position adjustment were measured according to a previous method [41]. The reference plane was demarcated on the CT images. The sagittal section passing through the centre of the horizontal axis of the condyle on the MRI data was used as the measurement reference plane, on which the projection of the reference plane determined the X- and Z-axes (Fig. 5a). The amounts of condylar and disc movements were measured along the X- and Z-axes, respectively. The condylar position shift was defined as the displacement of the condylar point measured with the condylar point before adjustment as the origin (Fig. 7a). Disc position shift was defined as the displacement of the posterior margin of the disc (disc point) (Fig. 7b). The condylar point and the disc point were joined to form a straight line, and the angles between the line and the Z-axis before and after adjustment were measured to evaluate the disc-condyle relationship (Fig. 7c).

### 2.5 Statistical analysis

Measurements were statistically analysed using IBM SPSS Statistics (v.22; IBM Corp, Chicago, IL) (α = 0.05). Shapiro–Wilk and Levene tests were used to evaluate the normality of the grouped data and equivalence for variance, respectively. Descriptive statistics, including mean, standard deviation, median, minimum (min), and maximum (max) values, were established for analysis. An independent *t*-test was performed to evaluate the shift in the positions of the condylar point and ipsilateral condylar ball point along the Y-axis and X- and Z-axes after adjusting for the IP variable. The Mann–Whitney *U* test and Kruskal-Wallis test were applied to assess the statistical differences in measurements for accuracy and repeatability, the amount of condylar position shift, and the disc-condyle angle, as the remaining data were not normally distributed. Internal error analysis for the two experimenters using the conventional method was performed using the intraclass correlation coefficient (ICC) to estimate the reliability and accuracy of the conventional method. Pearson and Spearman correlation analyses were used to investigate the interactions between the amount of disc position shift, disc morphology, and the amount of condylar position shift.

## 3 Results

### 3.1 Comparison of occlusal transfer repeatability

**Fig. 8:**
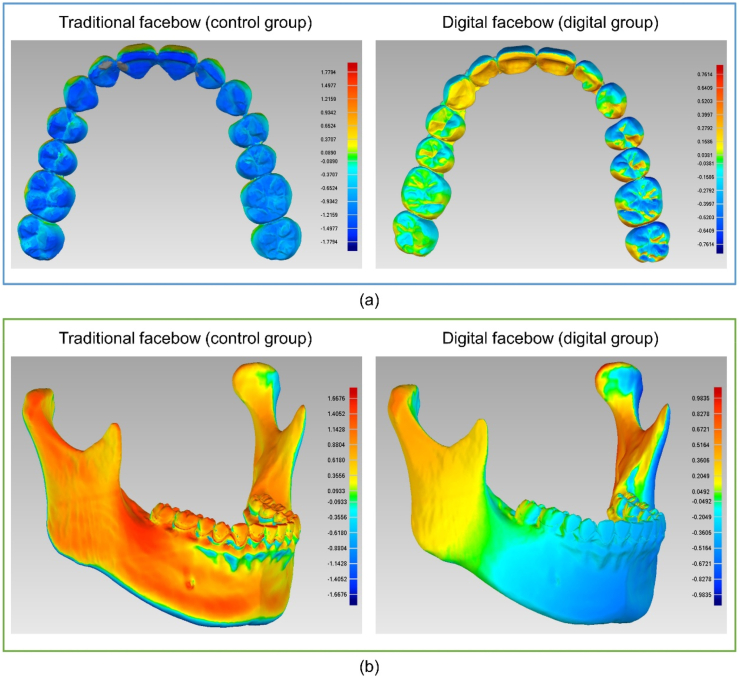
Spatial difference in the maxillary dentition (a) and the mandible (b) using different transfer methods. The right side of each graph represents the margin of error shown in colour.

The repeatability of the results obtained using the two facebow transfer methods was evaluated. Spatial differences in the maxillary dentition and mandible are shown as colour deviations (Fig. 8a and b).

The centroid offsets and RMS comparisons of the maxillary dentition and mandible as well as the bilateral condylar point offsets are listed in Table 1. The digital group had a significantly lower deviation in all comparisons than the control group (p < 0.001).

**Table 1:** Deviations in each measurement between the two groups (unit: mm).

Classification of variables	Groups	N	Mean	SD	Median	Min	Max	p
Centroid of the maxillary dentition	Control	30	1.71	1.12	1.43	0.39	4.77	
	Digital	30	0.45	0.26	0.41	0.07	1.08	0.000*
RMS of the maxillary dentition	Control	30	0.96	0.64	0.82	0.20	2.66	
	Digital	30	0.27	0.13	0.24	0.07	0.57	0.000*
Left condylar point	Control	30	1.65	0.86	1.47	0.20	3.48	
	Digital	30	0.74	0.33	0.74	0.20	1.42	0.000*
Right condylar point	Control	30	1.71	0.74	1.53	0.74	3.35	
	Digital	30	0.84	0.45	0.90	0.10	1.78	0.000*
Centroid of the mandible	Control	30	1.59	1.00	1.34	0.37	4.34	
	Digital	30	0.47	0.26	0.46	0.07	1.12	0.000*
RMS of the mandible	Control	30	0.91	0.48	1.43	0.39	4.77	
	Digital	30	0.34	0.14	0.41	0.07	1.08	0.000*

N: number, SD: standard deviation, RMS, root mean square. *p ＜0.05.

The consistency (95 % confidence interval) between the two operators was 0.947 when occlusal transfers were performed using the conventional method, confirming the reliability of the operations.

### 3.2 Comparison of condylar position adjustment accuracy

There was a difference in the positional deviation between the right condylar point and the ipsilateral condylar ball point after using the two workflows. Along the X-(p ＜0.05) and Z- (p ＜0.001) axes, the positional deviation in the digital group was significantly lower than that in the control group, while no obvious difference was found along the Y-axis (p > 0.05) (Table 2).

**Table 2:** Variable values of positional deviation between the right condylar point and ipsilateral condylar ball point during articulation for the two workflows.

Classification of variables	Groups	N	Mean	SD	Median	Min	Max	p
X	Control	15	2.95	1.74	2.46	0.28	6.63	0.010*
	Digital	15	1.43	0.80	1.29	0.06	2.54	
Y	Control	15	2.87	1.14	2.51	1.42	5.01	0.600
	Digital	15	3.06	0.86	3.38	1.28	4.36	
Z	Control	15	6.47	4.32	5.70	0.87	12.07	0.000*
	Digital	15	0.65	0.37	0.65	0.06	1.38	

N: number, SD: standard deviation. *p ＜0.05.

**Fig. 9:**
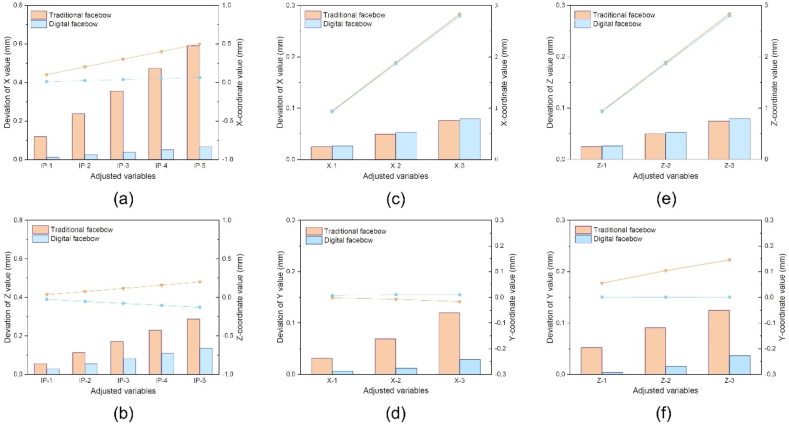
The bar chart shows the coordinate deviation between the right condylar point and the ipsilateral condylar ball point after adjusting for different variables in the control (orange) and digital (blue) groups. The line graph shows the trend of the coordinate value of the right condylar point with different variables in the control (orange) and digital (blue) groups.

Based on the transfer results, adjusting the IP variable caused positional deviation of the right condylar point and the ipsilateral condylar ball point along the X- and Z-axes (Fig. 9a and b). As the IP increased, the positional deviation in both groups increased accordingly. The digital group showed a significantly lower dispersion than the control group. Increasing the translation along the X-or Z axes on one side (illustrated on the right side) rendered similar results without significant differences between the digital and control groups (Fig. 9c and e). In contrast, a significant difference was found along the Y axis, and the digital group showed a lower deviation and dispersion than the control group (Fig. 9d and f).

### 3.3 Measurement and analysis of clinical cases

Of the 72 TMJs, 19 joints had a normal disc-condyle position, 43 had disc displacement with reduction (DDWR), and 10 had disc displacement without reduction (DDWoR) [42]; 41 discs were recognised as class 1, 16 as class 2, and 15 as class 3 [35].

**Fig. 10:**
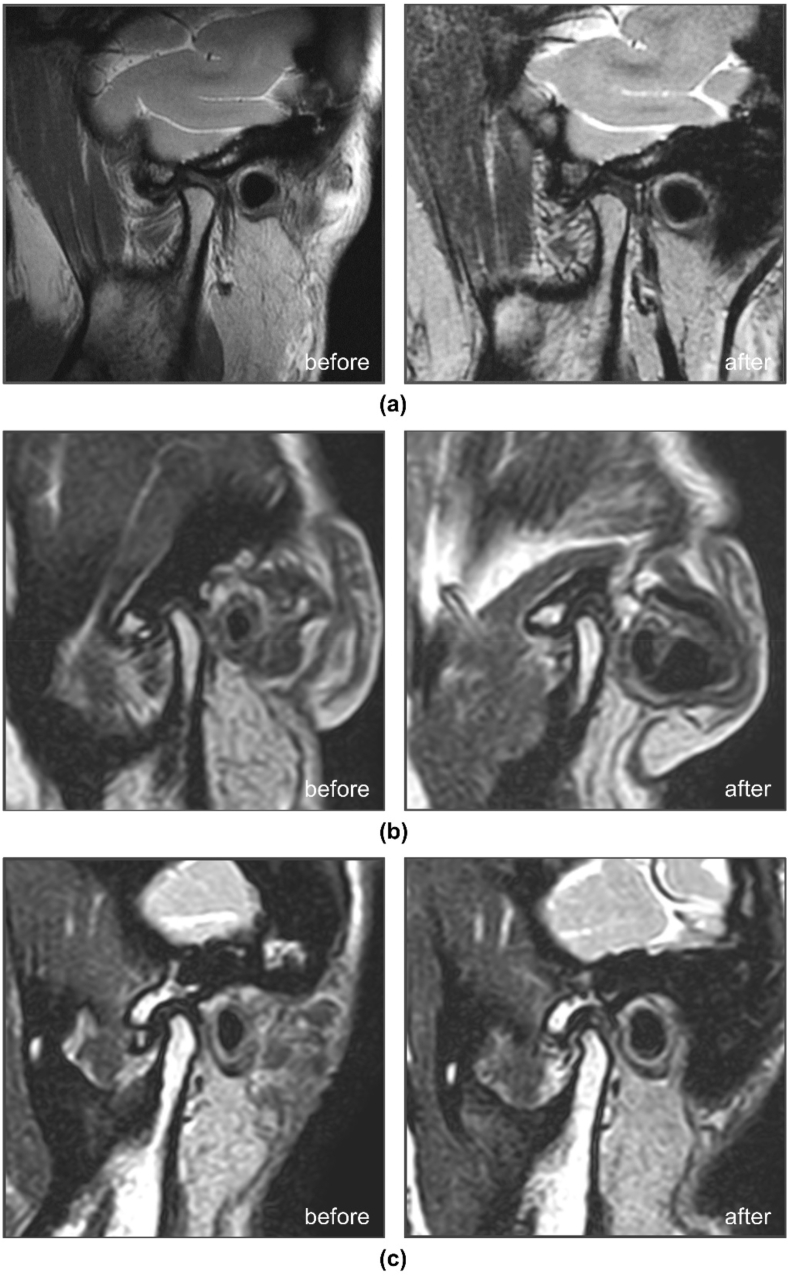
Partial magnified magnetic resonance imaging scan, including the area of temporomandibular joint. The diagrams on the left and right of each group represent the scans before and after Disc displacement with reduction (DDWR) with class 1 disc → Normal position with class 1 disc. (b) DDWR with class 2 disc → Normal position with class 1 disc. (c) DDWR with class 3 disc → Normal position with class 1 disc.

The amount of condyle and disc position shift before and after condylar position adjustment along the X- and Z-axes are shown in Table 3, and the disc-condyle angles before and after adjustment are shown in Table 4. Fig. 10a–c shows the MRI scans of representative cases with changes in the disc-condyle relationship and disc morphology before and after adjustment.

**Table 3:** Amount of condyle and disc position shift along X and Z axes before and after condylar position adjustment (mm, mean ± standard deviation).

Groups	X-coordinate			p	Z-coordinate			p
	Condyle	Disc	Correlation		Condyle	Disc	Correlation	
Normal position	0.86 ± 0.74	−0.28 ± 0.55	0.216	0.375	0.60 ± 0.64	0.59 ± 0.55	0.501	0.029*
Disc displacement with reduction	1.71 ± 1.12	−1.37 ± 1.50	−0.323	0.035*	1.01 ± 0.74	−0.09 ± 1.08	−0.050	0.752
Disc displacement without reduction	2.56 ± 1.81	0.68 ± 1.35^b^	0.057	0.875	0.61 ± 1.15	0.37 ± 1.45	0.276	0.074

*p < 0.05.

**Table 4:** Disc-condyle angle before and after condylar position adjustment (°, mean ± standard deviation).

Groups	Disc-condyle angle		p
	Before adjustment	After adjustment	
Normal position	−17.83 ± 11.48	−30.20 ± 12.96	0.008*
Disc displacement with reduction	39.66 ± 27.62	−2.35 ± 32.00	0.000*
Disc displacement without reduction	89.01 ± 14.67	76.91 ± 27.47	0.326

*p < 0.05.

For normal TMJs, the condyle moved 0.86 mm forward and 0.60 mm downward after condylar position adjustment, and the disc moved 0.28 mm backward and 0.59 mm downward, with the average disc-condyle angle reduced from −17.83° before adjustment to −30.20° after adjustment. The amount of disc position shift showed strong correlations with the amount of condylar position shift along the Z-axis, and the amount of angular variation showed strong correlations with the amount of condylar position shift along the X-axis.

For the joints with DDWR, the condyle moved 0.61 mm forward and 1.01 mm downward, and the disc moved 1.37 mm backward and 0.09 mm upward, with the average disc-condyle angle reduced from 39.66° to −2.35°. The amount of disc position shift showed a moderate correlation with the amount of condylar position shift along the X-axis, and the amount of angular variation showed a strong correlation with the amount of condylar position shift along the X- and Z-axes.

For the joints with DDWoR, the condyle moved 2.56 mm forward and 1.01 mm downward, the disc moved 0.68 mm forward and 0.37 mm downward, and the average disc-condyle angle before and after condylar position adjustment were 89.01° and 76.91°. The degree of disc position shift and– the disc-condyle angle showed little correlation with the condylar position shift along the X- and Z-axes.

For class 1 discs, there was no alteration in the disc shape after adjustment. For class 2 discs, the overall improvement rate was 56.25 % (9/16), and the changes in disc morphology showed strong correlations with the shift in condylar position along the X-axis. For class 3 discs, no significant differences in the changes in disc morphology were found with the amount of condylar position shift.

## 4 Discussion

Intervention in occlusion and jaw position improves TMJ overload [43,44]. Sound and reliable occlusal transfer is a prerequisite for effective condylar position adjustment and influences treatment outcomes [3,41,45]. The current study introduced a novel digital technique that enables real-time visualisation of occlusal transfer and adjustment of condylar position. In this study, quantitative analysis using RMS and positional shift measurements showed that the digital group had better repeatability, which is consistent with the assumptions. In addition, an ICC evaluation of the transfer results between two experienced experts using the conventional method suggested that the errors were not caused by the operation itself. After occlusal transfer using the digital method, the position errors between tests that adjusted the condylar position based on the results of each transfer were smaller, particularly along the X- and Z-axes, indicating that the transfer and adjustment using the new method improved the accuracy. This is consistent with the results of a previous study in which errors in facebow transfers based on 3D analysis were mainly influenced in the sagittal dimension [46].

In the condylar position adjustment method, the X and Z axes are the major directions of articular disc displacement and condylar position adjustment treatment, which means that inaccuracies in occlusal transfer using conventional methods may affect clinical treatment outcomes [47,48].

The differences in the condylar position shift between the two methods in this study were compared after adjusting for the direction of rotation (IP) and translation (X and Z) in the CPV. For rotation, the offset was larger when the conventional method was used and increased with the amount of adjustment. For translation, the difference was mainly observed in the direction perpendicular to the direction of adjustment. Therefore, conventional occlusal transfer may be used to translate the condyle in the sagittal direction according to the plan, but it introduces errors in other directions. Discrepancies in design and results result in unfavourable clinical outcomes.

Regarding the positioning of the condyle in the coronal direction along the Y-axis, no significant difference was detected between the two transfer methods; however, a difference was observed after adjusting the condylar position.

The digital workflow showed a preferable performance owing to its precise definition of reference points based on an individualised stomatognathic bony framework. Adjustment is directly related to the condyle, helping avoid errors caused using superficial anatomical landmarks to achieve better repeatability and precision [16,17,49,50].

In this study, the results of the analysis of in vivo measurements showed that the joint structure was significantly improved, and the condyles moved forward and downward in all groups after adjusting the condylar position using the digital method. In the DDWR group, the articular disc returned backward and upward with the condyle, whereas in the DDWoR group, changing the position of the condyle resulted in subsequent forward and downward movement of the articular disc. Overall, the improvement rate in the disc-condyle relationship was 93.06 % (67/72), which achieved better adjustment results than the results of the disc-condyle relationship after pre-existing MRI-guided adjustment of the jaw position (84.62 % improvement rate in the DDWR group) [5]. In this study, the results showed that all the patients’ clinical symptoms improved to some extent, with the most significant improvement in the symptoms of abnormal joint sounds and joint pain. After adjusting the condylar position, clicking symptoms or crepitus sounds in the TMJs were relieved in 67.44 % (29/43) of the patients, and 87.50 % (7/8) of the patients no longer experienced pain during movement. This finding demonstrated that the novel digital condylar position adjustment technique was therapeutically feasible for patients with TMD.

The available findings confirm the advantages of this digital workflow in terms of repeatability and accuracy and provide an initial demonstration of the clinical effectiveness of condylar adjustment using digital methods. Compared to the previously introduced MRI-guided articulating technique [5], the current method lowers the dependence on sophisticated equipment and increases efficiency. In addition, the novel digital technique allows for real-time visualisation of condylar position adjustment. However, a limitation of this study is the lack of comparison of bilateral condylar position adjustments in patients with facial asymmetry. Errors in adjusting the condylar position bilaterally after facebow occlusal transfer should also be considered. Digital-technology-based plans offer reliable and promising alternatives. Further research and development would facilitate the optimisation of the process. In combination with updated visualisation techniques and artificial intelligence, precise control of the condylar position is expected.

## 5 Conclusion

This study introduces a novel digital workflow that is repeatable and accurate. By combining CT data with digital dental casts, precise adjustment of the condylar position can be achieved, which is helpful in restoring the disc-condyle relationship. This new method can be an effective alternative to conventional methods by simplifying cumbersome steps while reducing the number of uncontrollable variables that can lead to errors.

## Ethical approval

This study was reviewed and approved by the Institutional Review Board of West China Hospital of Stomatology (approval number: WCHSIRB-D-2022-208-R1). All participants (or their proxies/legal guardians) provided informed consent to participate in the study and for the publication of their anonymised case details and images.

## Data availability statement

Data will be made available on reasonable request.

## CRediT authorship contribution statement

**Yanji Gong:** Writing – review & editing, Writing – original draft, Validation, Investigation, Formal analysis, Conceptualization. **Fang Liu:** Writing – review & editing, Software, Methodology. **Yunfan Zhu:** Writing – review & editing, Software, Methodology. **Qinlanhui Zhang:** Writing – review & editing, Validation. **Jinyi Zhu:** Writing – review & editing. **Yang Liu:** Supervision, Project administration, Funding acquisition, Conceptualization. **Deqiang Yin:** Supervision, Project administration.

## Declaration of competing interest

The authors declare the following financial interests/personal relationships which may be considered as potential competing interests:Yang Liu has patent #2022SR0077807 licensed to Yang Liu. If there are other authors, they declare that they have no known competing financial interests or personal relationships that could have appeared to influence the work reported in this paper.
